# Changes in Brain Network Efficiency and Working Memory Performance in Aging

**DOI:** 10.1371/journal.pone.0123950

**Published:** 2015-04-13

**Authors:** Matthew L. Stanley, Sean L. Simpson, Dale Dagenbach, Robert G. Lyday, Jonathan H. Burdette, Paul J. Laurienti

**Affiliations:** 1 Laboratory for Complex Brain Networks, Wake Forest School of Medicine, Winston-Salem, North Carolina, United States of America; 2 Department of Biostatistical Sciences, Wake Forest School of Medicine, Winston-Salem, North Carolina, United States of America; 3 Department of Psychology, Wake Forest University, Winston-Salem, North Carolina, United States of America; 4 Department of Radiology, Wake Forest School of Medicine, Winston-Salem, North Carolina, United States of America; Beijing Normal University,Beijing, CHINA

## Abstract

Working memory is a complex psychological construct referring to the temporary storage and active processing of information. We used functional connectivity brain network metrics quantifying local and global efficiency of information transfer for predicting individual variability in working memory performance on an *n*-back task in both young (*n* = 14) and older (*n* = 15) adults. Individual differences in both local and global efficiency during the working memory task were significant predictors of working memory performance in addition to age (and an interaction between age and global efficiency). Decreases in local efficiency during the working memory task were associated with better working memory performance in both age cohorts. In contrast, increases in global efficiency were associated with much better working performance for young participants; however, increases in global efficiency were associated with a slight decrease in working memory performance for older participants. Individual differences in local and global efficiency during resting-state sessions were not significant predictors of working memory performance. Significant group whole-brain functional network decreases in local efficiency also were observed during the working memory task compared to rest, whereas no significant differences were observed in network global efficiency. These results are discussed in relation to recently developed models of age-related differences in working memory.

## Introduction

Substantial progress has recently been made in the neurosciences by investigating the structure and function of the brain as a large-scale complex network [1–2]. All complex networks fundamentally consist of two basic components: differentiable elements of the system and the pairwise relationships between those elements. Formally, these elements are represented as nodes, and the pairwise relationships between elements are represented as edges. In human functional brain networks, nodes represent a predefined collection of brain tissue, and edges represent measured functional connectivity between pairs of nodes. Graph theory measures allow for the quantitative characterization of complex patterns of organization within and between large-scale brain networks. The ability to analyze these networks with graph theory measures affords a biologically meaningful research program capable of identifying critical changes in certain network properties in both normal and disordered cognitive function across the life span. The primary purpose of this paper is to explore the utility of functional brain network measures of information transfer in accounting for individual variability in working memory performance on an *n*-back task in both young and older adults.

Working memory broadly refers to the simultaneous temporary storage and active processing of information in the service of goal-directed behaviors [3]. Numerous studies have measured working memory performance across the adult life span, consistently and reliably reporting that working memory performance exhibits a gradual and regular decline from early to late adulthood [4–8], presumably due to widespread decreases in neural and metabolic efficiency resulting from molecular, cellular, and structural changes in the aging brain [9]. Studies using fMRI to investigate the effects of aging on patterns of functional activation associated with working memory have primarily focused on localizing and functionally characterizing changes in certain brain regions that facilitate different components of the working memory construct [10–15]. However, approaching the study of the brain as an adaptive system with various interdependent, interacting components that give rise to complex behaviors may confer additional insights not obtainable from measuring anatomically localized and encapsulated activations [16]. In fact, rapidly accumulating evidence using multivariate methodologies to explore functional connectivity patterns has suggested that integrative processes and dynamic, complex interactions across numerous, distributed brain regions subserve visual recognition [17], language functions [18], cognitive control and executive functioning [19], emotion-cognition interactions [20], decision making processes [21], and social cognition [22]. Despite this recent emphasis on integrative processes and complex interactions between distributed brain regions for the study of cognition, no studies have been conducted to investigate age-related differences in complex patterns of functional connectivity associated with working memory performance.

Most published work investigating the topology of complex brain networks has examined differences in resting state functional connectivity (rs-fcMRI) across certain groups of interest as opposed to patterns of complex network topology during cognitive tasks. rs-fcMRI analyses measure correlations in spontaneous low-frequency fluctuations in blood oxygen level dependent (BOLD) signal in participants while participants are engaged in internally oriented mental activities in the absence of externally focused goal-directed tasks [23–24]. This contrasts with task-evoked fMRI analyses that capture transient activations or deactivations over several seconds when a participant is presented with an external stimulus or engaged in some cognitive task. The temporal coherence in neural activity measured in rs-fcMRI data is taken to reflect underlying, intrinsic patterns of neural activity and has provided evidence of both functional homogeneity in adjacent brain areas and functional connectivity between distant, noncontiguous brain areas [23, 25–29]. A considerable quantity of work has focused on these patterns of intrinsic functional connectivity in rs-fcMRI data, which appear to be strongest within and between functionally related brain areas [30–31]. That is, these patterns are strongest among brain areas that are similarly modulated by various task paradigms [32–33].

Results from recent graph theoretic functional brain network analyses have demonstrated that differences in certain topological properties of intrinsic resting-state connectivity patterns are predictive of intelligence [34], working memory capacity [35], verbal learning and visual recall [36], normal aging [37–40], and certain neurological disorders including Alzheimer’s disease [41–42], Parkinson’s disease [43] and schizophrenia [44–45]. Although less is known about functional network topology during active experimental task performance, it is important to examine functional brain network properties during diverse tasks because certain topological properties present during rest may change during tasks. In fact, non-trivial differences in functional brain network topology have recently been identified during auditory and visual stimulation [46], odor recognition memory tasks [47], motor learning [48–49], decision making processes [21], emotional face processing [50], and changes in working memory load [51].

Of particular relevance to the current study, Rzucidlo et al. (2013) recently demonstrated that mean group values of whole-brain network local efficiency tend to decrease from resting-state to a commonly used working memory task, the *n*-back task. However, the same study reported that mean group values of whole-brain network global efficiency were not significantly different across conditions [52]. The topological organization of a network is directly related to its local and global efficiency, which jointly determine the network’s capability of integrating information effectively [1–2, 53]. Local efficiency provides an indication of how effectively information is integrated between the immediate neighbors of a given network node, whereas global efficiency provides an indication of how effectively information is integrated across the entirety of the network. In the current study, we extend those analyses conducted by Rzucidlo et al. (2013) by determining whether the change in mean group values of whole-brain network local and global efficiency from rest to the *n*-back task differ between young and older adults. We further extend the work of Rzucidlo et al. (2013) by exploring the utility of local and global efficiency in accounting for individual variability in working memory performance across age groups.

### Research Aims

The primary purpose of the current study is to investigate the utility of whole brain network metrics across age groups (young and older adults) in accounting for individual differences in working memory performance. More specifically, we sought to determine (1) whether local and global efficiency are predictive of working memory performance; (2) whether local and global efficiency predict working memory performance differentially in young and older participants; and (3) whether local and global efficiency are differentially predictive of working memory performance at rest and during task. Secondarily, we sought to investigate potential differences between mean group measures of local and global efficiency from resting-state to the working memory condition and between age groups.

## Materials and Methods

### Ethics Statement

The research protocol was reviewed and approved by the Wake Forest School of Medicine Institutional Review Board (IRB). Informed consent was obtained in writing from each participant following the protocol approved by the IRB.

### Participants

Fifteen older adults (11 females, *M*_*age*_ = 72.07 years, *SD* = 3.47, age range: 66–79 years) and 14 younger adults (9 females, *M*_*age*_ = 27.21 years, *SD* = 4.00, age range: 22–34 years) participated in this study. All participants were recruited via locally placed advertisements followed by a telephone screening. Participants were included only after fulfilling several criteria on batteries for cognition, including the Center for Epidemiological Studies Depression Scale (CES-D; [54]) and the Modified Mini-Mental State Examination [55–56]. Participants scoring greater than or equal to 25 of the CES-D were excluded from the study. Additionally, only right-handed participants with functional color vision [57], no history of alcoholism (AUDIT; [58]), corrected visual acuity, and no more than moderate hearing loss were included in these analyses. Participants were originally recruited as controls in three separate Body Mass Index (BMI) groups—normal weight (BMI from 18.5 to <25), overweight (BMI from 25 to <30), and obese (BMI from 30 to <40)—as part of a larger parent study investigating the effects of aging and obesity on the brain. No systematic effects of BMI group were observed within or between age groups or conditions. As such, we combined all BMI groups for the analyses herein.

### Scanning Procedure

During each scanning session, fMRI data were acquired during rest and the *n*-back task with *n* = 2. All participants were provided with fMRI compatible goggles, ear plugs, and a hand-held button box custom-made to be MRI compatible and interfaced with the e-prime [59] response box (for the 2-back task) for the entirety of each session. Participants were instructed to keep their eyes open, not fall asleep, and fixate on a black cross on the computer screen interfaced with their goggles during rest. During the 2-back condition, a sequence of 100 letters was presented on the screen in the goggles. The order of the letters presented in the sequence differed between participants in order to minimize a potential systematic effect of the presentation sequence. Letters appeared for 0.3 seconds followed by a 2.7 second blank slide during which participants were asked to respond; as such, individual trials lasted for three seconds. The entire 2-back task lasted for a total of five minutes and 20 seconds. For every letter that appeared after the second letter, participants were required to determine whether the current letter was the same as the one presented two letters back in the sequence. The 2-back task requires the maintenance and continual updating of the contents of working memory as well as an executive component for keeping instructions and goals online while inhibiting incorrect, competing responses [60–61]. Participants were instructed to press one button on the hand-held box when the current letter was the same as the one presented two letters back in the sequence. Participants were instructed to press a different button on the hand-held box when the current letter was not the same as the one presented two letters back in the sequence. Before beginning the task, participants were instructed to respond as quickly and accurately as possible with their right hands using their index and middle fingers to differentiate responses.

### Data Acquisition and Pre-processing

For each participant, a multi-slice spoiled gradient inversion recovery (3DSPGR-IR) was used to collect high-resolution T_1_-weighted images on a 1.5T GE scanner. A GE 8 channel neurovascular headcoil was used. The protocol parameters were as follows: phase/frequency = 256/256; 156 contiguous slices, 1.0 mm thick; in-plane resolution of 0.938 mm x 0.938 mm; TE = 4.74 ms; TR = 4.68 ms; T1 = 600 ms. BOLD contrast was measured using a whole-brain gradient echo echo-planar imaging (EPI) sequence with the following parameters: phase/frequency = 64/64; 159 volumes with 28 contiguous slices per volume; slice thickness = 5.0 mm; in-plane resolution of 3.75 mm x 3.75 mm; TR/TE = 2000/40 ms.

Image preprocessing was performed using SPM8 software (http://www.fil.ion.ucl.ac.uk/spm/). All functional data were realigned, slice-time corrected, and co-registered to a skull-stripped version of the accompanying structural data. Coregistration was checked visually for each participant. Structural data were parcellated into gray matter, white matter, and cerebrospinal maps using the unified segmentation function in SPM8. As part of an integrated processing procedure, structural images were warped to MNI template space (Montreal Neurological Institute, http://www.mni.mcgill.ca/). The normalization parameters derived from the structural image warping were then applied to all functional data. A band-pass filter (0.009–0.08 Hz) was applied to remove physiological noise and low-frequency drift. Six rigid-body transformation parameters generated during the realignment process and three mean signals (whole-brain, white matter, and cerebrospinal fluid) were then regressed out of the functional data. Additionally, sinus midline and sinus occipital ROIs were regressed out of the functional data. All functional data were motion corrected to eliminate scan volumes with excessive frame-wise displacement and BOLD signal change [62]. Values of 0.5 for frame-wise displacement and 0.5% ΔBOLD for DVARS were chosen to represent values well above the norm found in still subjects. An average of 1.2 volumes were removed for young participants at rest; an average of 1.8 were removed for older participants at rest; an average of 0.5 were removed for young participants during 2-back; and an average of 2.1 were removed for older participants during 2-back. There were no significant differences in the number of volumes removed between age groups or within subjects from rest to the 2-back task (all *p*’s > .11). The number of volumes removed due to motion were also included in all regression analyses (see below) as a control.

### Generating Whole-Brain Networks

Pre-processed functional data were masked such that only gray matter voxels were included within areas specified by the Automated Anatomical Labeling (AAL) atlas [63]. We constructed brain networks by assigning a node to each voxel and then measuring correlations in activity computed from pairs of simultaneously recorded time series. Two separate networks were constructed for each participant: one network for resting state and another for the 2-back task. Using a voxel-wise approach to define nodes generates high resolution networks, unbiased and unconstrained by *a priori* assumptions that limit the potential for making new discoveries [64].

Voxel-wise functional brain networks were generated from a correlation matrix of time series data from each voxel pair using the Pearson correlation coefficient. Negative connections were not included in these analyses (for reasons noted in [16,39]). Edge density across subjects was matched using the formula *N* = *K* ^*S*^, with *N* equal to the number of nodes, *K* equal to the average degree, and *S* set at 2.0, 2.5, and 3.0 to assess any potential threshold effects. The same analyses were run at each threshold, and no results presented herein are threshold specific. Because prior work has demonstrated that brain networks tend to fragment when *S* is greater than three [65] and the reproducibility of brain networks is highest at thresholds between two and three [66], we present data using a threshold of *S* = 2.5. A correlation coefficient cut-off that meets this density threshold was determined and only those correlations above the threshold were considered as functional edges in the analyses presented herein. Those edges between any two given voxels that met the threshold requirement were given a value of one, and all other edges were given a value of zero. As such, undirected, unweighted adjacency matrices were generated for each participant representing whole-brain functional connectivity. Thresholding the network in this way ensures that comparisons are made between networks of comparable density relative to the total number of network nodes. Young and older participants had approximately the same number of nodes in all conditions, indicating that network measures between age cohorts were comparable.

### Network Metrics

#### Global efficiency

Global efficiency is a measure of the efficiency of distant information transfer in a network and is defined as the inverse of the average characteristic path length between all nodes in the network [67]. Beginning with each voxel representing a node and the similarity in the measured time series between any two voxels providing the basis for the existence of a functional connection, the shortest number of steps required to go from node *i* to every other network node was computed. This was done separately for each and every node in the network, and the average number of shortest steps to all other network nodes was computed separately for each node. The inverse of the average number of shortest steps for each node was then summed across all network nodes and this summed quantity is normalized by taking into account the total possible number of connections that could exist in the network. Formally, global efficiency is calculated as

$$E_{global}=\frac{1}{N(N-1)}\sum_{j,k\in G_{i}}\frac{1}{L_{j,k}}$$

where *N* is the set of all nodes in the network and *L*_*j*,*k*_ is the average distance (number of steps) between nodes *i* and *j* in the network. Global efficiency is a scaled measure ranging from 0–1, with a value of 1 indicating maximum global efficiency in the network. In functional brain networks, global efficiency provides a measure of the overall capacity for parallel information transfer and integrated processing among distributed components of the system [53]. Importantly, higher order cognitive functions, such as working memory, may require the integration of information from several disparate sources, benefiting from global efficiency across the entirety of the network [53].

#### Local efficiency

Local efficiency is a measure of the average efficiency of information transfer within local subgraphs or neighborhoods and is defined as the inverse of the shortest average path length of all neighbors of a given node among themselves [67]. Local efficiency was first computed for each individual node *i* in the network by identifying the set nodes, or subgraph, to which node *i* is directly connected. After removing node *i* from the identified subgraph, the shortest path between all nodes in the subgraph was calculated. The inverse of the shortest path from each node formerly connected to node *i* to every other node formerly connected to node *i* was then summed across all nodes formerly connected to node *i*, and this summed quantity is normalized by taking into account the total possible number of connections that could exist among all nodes formerly connected to node *i*. Formally, local efficiency is calculated as

$$E_{local}=\frac{1}{N_{G}{}_{{}_{i}}(N_{G}{}_{{}_{i}}-1)}\sum_{j,k\in G_{i}}\frac{1}{L_{j,k}}$$

where $N_{G}{}_{{}_{i}}$ represents the number of nodes in the subgraph *G*_*i*_. Local efficiency is a scaled measure ranging from 0–1, with a value of 1 indicating maximum local efficiency in the network. In functional brain networks, high local efficiency suggests a topological organization indicative of segregated neural processing [2]. The local efficiency of the network reveals how effectively information is transferred among the first neighbors of node *i* when node *i* is removed from the network. Nodes in networks with high local efficiency tend to effectively share information within their immediate local communities, which provides a basis for effective segregated information processing in the network.

### *d’* Measure of Working Memory Performance

Based on work in signal detection theory [68], the best available method for quantifying performance on the *n*-back task is a total score (*d’*) that takes into account the range for hits and false alarms by calculating the normalized proportion of correct hits minus the normalized proportion of false alarms. This measure of working memory performance is the dependent variable in all statistical modeling analyses herein. *d’* is calculated from the hit (H) rate and false-alarm (FA) rate using the formula *d*' = *Z*_*H*_ − *Z*_*FA*_, where *Z* represents a transformation of the two distributions to generate z-scores of the rate of hits and the rate of false alarms [69]. The better an individual maximizes hits (and thus minimizes misses) and minimizes false alarms (and thus maximizes correct rejections), the higher the individual’s *d’* score. Higher scores on the *d’* measure indicate better performance on the *n*-back task (for review of the *d’* measure, see [70]).

### Speed-Accuracy Trade-Off

Older adults tend to be substantially slower than young adults during most tasks that emphasize rapid responding (e.g., [71–72]). Two explanations for this age-related slowing in response times have gained traction in the literature. One explanation is that age-related increases in neural noise resulting from molecular, cellular, and structural alterations slow down all processes related to evidence accumulation and response selection at the same rate [73–77]. The second explanation is that older adults are simply more reluctant to commit errors and tend to attach a greater importance to responding accurately than to responding quickly [72, 78–80]. Thus, in order to avoid mistakes and achieve better performance, older adults are more likely to strategically balance the opposing demands of accuracy and speed, which is commonly referred to as the speed-accuracy trade-off [81]. In order to avoid this potential confound, we controlled for the possibility that older adults cautiously and strategically chose to accumulate more evidence before making a decision on the *n*-back task. Because the distributions of average response times (RT) on correct trials overlapped between young and older adults, exclusively including an age group variable was insufficient as a control. So, after excluding those responses +/- 3 *SD*s from the mean for each participant, the average RT on correct trials for each participant was recorded to serve as a control in regression analyses.

### Statistical Analyses

Age-related group differences in local and global efficiency were computed using independent samples *t*-tests, while paired-samples *t*-tests were used to identify potential differences in local and global efficiency between resting state and the 2-back task within subjects.

In order to determine whether differences in local and global efficiency account for a substantial proportion of individual variability in working memory performance across age groups, backward/forward stepwise linear regression analyses were conducted with Akaike’s information criterion (AIC; [82–83]) and Adjusted R^2^ as criteria to uncover the model that accounts for substantial individual variability in working memory performance. AIC provides a measure of the quality of a statistical model by simultaneously maximizing the goodness of fit of the model (amount of variance explained) while minimizing the number of parameters included in the model. With AIC and Adjusted R^2^ as criteria, backward/forward stepwise linear regression is a semi-automated process of successively removing or adding variables to identify the model that simultaneously ensures that the goodness of fit of the model is maximized while minimizing the complexity of the model. Age was included as an independent variable and coded as a binary, categorical variable (0 = young, 1 = old). *d’* was used as the dependent variable in all regression analyses. We sought to quantify the relationship between working memory performance and each of the covariates, all possible interactions between those covariates, and all possible quadratic terms after controlling for the variability in working memory performance explained by the speed of responding (measured in milliseconds) and the number of volumes removed per participant due to excessive motion.

## Results

### Behavioral Results

The mean *d’* score for younger participants was 3.44 (*SD* = 1.01), and scores ranged from 1.69 to 4.61; the mean *d’* score for older participants was 2.15 (*SD* = .72), and scores ranged from. 84 to 3.35. Working memory performance (measured by *d’*) on the 2-back task was significantly worse among older participants than younger participants, *t*(27) = 4.00, *p* < .001, Cohen’s *d* = .74.

### Whole Brain Mean Differences in Local and Global Efficiency

For each participant, whole-brain network measures of local and global efficiency were computed during resting-state and the working memory task for each participant. These values were then averaged within each age group to obtain group means, and standard deviations of the means across subjects were computed (Table 1).

**Table 1:** Mean (SD) local and global efficiency for both resting and task states between age groups.

*Condition*	*Young Adults (n = 14*, *48%)*		*Older Adults (n = 15*, *52%)*	
	*E*_*local*_	*E*_*global*_	*E*_*local*_	*E*_*global*_
**Rest**	0.447 (.03)	0.243 (.03)	0.436 (.04)	0.243 (.04)
**2-back**	0.411 (.02)	0.251 (.03)	0.413 (.04)	0.236 (.04)

No significant differences were obtained between age groups during rest for local efficiency *t*(27) = .88, *p* = .39, or global efficiency *t*(27) = .03, *p* = .98. No significant differences were obtained between age groups during the 2-back task for local efficiency, *t*(27) = .15, *p* = .88, or global efficiency, *t*(27) = 1.22, *p* = .23. However, there was a significant difference in the change in local efficiency from rest to 2-back with the groups combined, *t*(28) = 4.00, *p* < .001, Cohen’s *d* = .74. Post-hoc paired-samples *t*-tests for each age group indicated that there was a significant difference in local efficiency from rest to 2-back for young participants, *t*(13) = 4.22, *p* = .001, Cohen’s *d* = 1.13, and a marginally significant difference for older participants, *t*(14) = 1.96, *p* = .070, Cohen’s *d* = 0.51. No significant differences in global efficiency were obtained across conditions in either age group.

### Working Memory Performance and Brain Network Efficiency

The primary purpose of this study is to determine whether differences in local and global efficiency account for a substantial proportion of individual variability in working memory performance across age groups. In order to approach this research question, we conducted backward/forward stepwise linear regression analyses with Akaike’s information criterion (AIC; [82–83]) and Adjusted R^2^ as criteria to discover (1) whether individual differences in local and global efficiency are significantly predictive of working memory performance, and (2) whether local and global efficiency are dependent upon age-related differences in predicting working memory performance. Age was included as an independent variable and coded as a binary, categorical variable (0 = young, 1 = old). *d’* was used as the dependent variable in all regression analyses. We sought to quantify the relationship between working memory performance and each of the covariates, all possible interactions between those covariates, and all possible quadratic terms while controlling for the variability in working memory performance explained by the speed of responding (measured in milliseconds) and the number of volumes removed per participant due to excessive motion.

Local efficiency during task, global efficiency during task, average response time, number of volumes removed due to motion, participant age group, and all possible interactions and quadratic terms were assessed in identifying the most efficient model for predicting working memory performance. After completing backward/forward stepwise regression with AIC and Adjusted R^2^ as criteria, the most efficient model that emerged accounted for 73% of the variance in working memory performance as measured by *d’* scores. The predictors in the final model were: local efficiency (*b* = -9.46, *p* = .038), global efficiency (*b* = 20.29, *p* = .005), average response time (*b* = -.004, *p* < .001), age (*b* = 5.53, *p* = .011) and an interaction between global efficiency and age (*b* = -23.04, *p* = .008). The Adjusted R^2^ value was. 671, the model was significant (*F*(5,23) = 12.42, *p* < .001), and the model produced an effect size of 2.704 (Cohen’s *f* ^2^), collectively indicating that the model is highly effective in explaining individual variability in working memory performance across age groups. Evaluation of condition indices and variance inflation factors showed no issues with multicolinearity. Evaluation of residual plots showed that the conditions of linearity, normality, and constant variance were satisfied. Thus, all conditions were satisfied for the linear model. Importantly, the number of volumes removed due to motion did not significantly predict working memory performance. Table 2 provides a summary of the final model.

**Table 2:** Summary of regression analyses for variables predicting working memory performance with d’ (*N* = 29).

Variable	*b*	*SE*	*t*	*p*	*95% CI*
Local Efficiency	-9.461	4.288	-2.207	0.038*	[-18.330, -0.591]
Global Efficiency	20.290	6.565	3.091	0.005**	[6.712, 33.874]
Average Response Time	-0.003	0.001	-4.383	0.000***	[-0.005, -0.002]
Age	5.534	2.012	2.750	0.011*	[1.372, 9.696]
Global Efficiency x Age	-23.044	7.920	-2.910	0.008**	[-39.427, -6.662]
*Adjusted R* ^*2*^				0.671	
*F-statistic*				12.42***	

*p < .05.

**p < .01.

***p < .001

The final model obtained was then deconstructed into separate regression equations for young and older cohorts. The model with unstandardized coefficients for the young age group was:

$$\hat{d}{'}_{young}=3.6708-9.4607\cdot E_{local}+20.2928\cdot E_{global}-0.0034\cdot RT$$

In contrast, the model with unstandardized coefficients for the older age group was:

$$\hat{d}{'}_{older}=9.2047-9.4607\cdot E_{local}-2.7517\cdot E_{global}-0.0034\cdot RT$$

**Fig 1:**
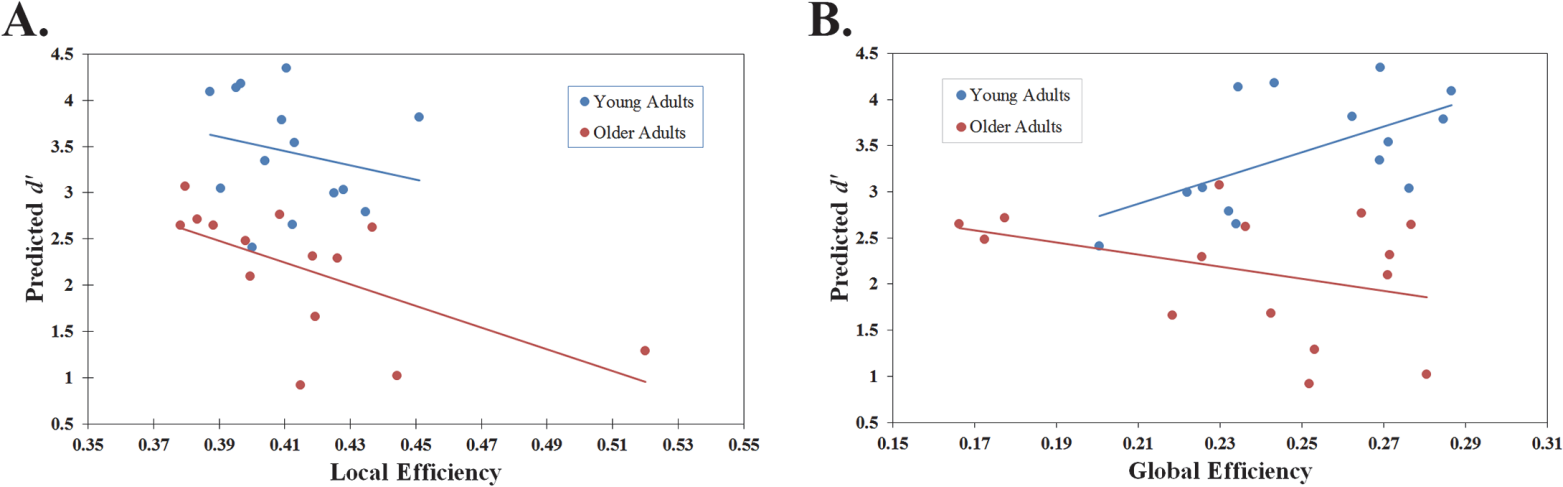
Graphical Summary of Results from Final Regression Model. The predicted *d’* values from the final model (the model containing local efficiency during task, global efficiency during task, age group, average RT, and an interaction between global efficiency during task and age group as parameters) are plotted against observed local efficiency (A) and global efficiency (B) values during the 2-back task, respectively, and split by age group.

Although the predictive utility of local efficiency remained the same for both age groups, both the magnitude and direction of the global efficiency variable differed between young and older adults for predicting working memory performance. This demonstrates that slight increases in global efficiency among young adults produce substantial improvements in working memory performance, whereas the same magnitude increases in global efficiency among older adults produce slight decreases in working memory performance. Fig 1 provides a graphical summary of the results from the final regression model.

In a second model, local efficiency *during resting-state*, global efficiency *during resting-state*, average response time, number of volumes removed due to motion, participant age group, and all possible interactions and quadratic terms were assessed in identifying the most efficient model for predicting working memory performance. After completing backward/forward stepwise regression with AIC and Adjusted R^2^ as criteria, neither local nor global efficiency at rest were significant predictors of working memory performance, and thus, will not be discussed further in this section.

## Discussion

The primary purpose of this study was to analyze the relationship between the efficiency of information transfer in the brain and working memory performance in both young and older adults. We have demonstrated that these biologically meaningful whole-brain measures account for a considerable proportion of the variance in working memory performance. Both local and global efficiency during the *n*-back task were significant predictors of working memory performance in addition to age (and an interaction between age and global efficiency), even after controlling for potential age-related and within group differences in the speed-accuracy trade-off. After identifying the best model using AIC and Adjusted R^2^ as criteria, the results indicated that decreases in local efficiency during task were associated with better working memory performance in both age cohorts. In contrast, the results also indicated that increases in global efficiency during task were associated with much better working performance for young participants; however, increases in global efficiency during task were associated with a slight decrease in working memory performance for older participants. Thus, changes in global efficiency during the *n*-back task were differentially important in accounting for individual variability in working memory performance between age groups. However, neither local nor global efficiency at rest were significant predictors of working memory performance.

Furthermore, we found that mean whole-brain measures of local efficiency decreased significantly in young adults during 2-back compared to rest, akin to what was observed by Rzucidlo et al. (2013), and marginally significantly in older adults. There was also a substantial difference in the magnitude of the effect size between young and older participants from rest to 2-back for the local efficiency measure. Whereas the change in local efficiency from rest to 2-back among the younger adults was quite substantial, the older adults only displayed a moderate shift. No whole-brain mean differences in global efficiency were observed for either age group between rest and 2-back. No age-related differences in local or global efficiency were observed within conditions either.

Lower local efficiency during the working memory task was significantly associated with better working memory performance within both age groups. The local efficiency measure has traditionally served as an indication of the degree to which segregated (or specialized) information processing persists in the brain. The idea that localized brain regions are functionally specialized information processing units and make specific contributions to cognitive processes is supported by a substantial body of evidence from activation studies. However, localized functional specialization alone cannot fully account for most aspects of brain function [84]. Integrated (or distributed) processes (e.g., executive functions commonly included in the working memory construct) may instead benefit from high global efficiency of information transfer across the brain as a whole, especially for more demanding tasks that require more complex operations for successful performance [53]. For example, van den Heuvel et al. (2009) demonstrated that higher IQ scores are associated with greater global efficiency in functional brain networks. In line with van den Heuvel et al. (2009), our results demonstrate that while local functional specialization does not facilitate better behavioral performance, greater integration of information across the network, measured with global efficiency, is associated with better working memory performance at least for young adults.

Functional brain networks tend to exhibit small-world properties traditionally characterized by high clustering and low path length, which jointly support the efficient transfer of parallel information at a relatively low cost [85–89]. High clustering supports the functional specialization of local collections of densely interconnected nodes. Shorter path lengths ensure that information easily spreads throughout the network, making parallel and distributed information processing possible. Extensive research has established that short path length is a characteristic of random networks, whereas high clustering is a property of lattice networks [90]. Small-world networks possess clustering comparable to a regular lattice and path length comparable to a random network. Because local efficiency closely corresponds to clustering and global efficiency is computed as the inverse of path length, recent work has explored small-world properties using local and global efficiency [37, 67, 91]. However, little work has explored whether small-world properties in functional brain networks facilitate better behavioral performance on cognitive tasks. For it remains possible that more clustered than distributed brain networks during tasks facilitate better behavioral performance or that more distributed than clustered brain networks during tasks facilitate better behavioral performance. Among young adults, increases in global efficiency accompanied decreases in local efficiency for predicting better working memory performance. This suggests that a more distributed than tightly clustered network facilitates better working memory performance.

Furthermore, several previous studies have demonstrated that average group whole-brain local efficiency during resting-state decreases from early to late adulthood [37, 39–40]. However, we observed no significant differences in local efficiency between age groups. This difference between our study and previous work could be due to differences in nodal definition, which can dramatically impact network metrics [65, 92]. These three previous studies utilized a more macroscopic nodal partitioning scheme, whereas we implemented a mesoscopic voxel-wise partitioning scheme. Because these networks were fundamentally constructed in such different ways, those results obtained from our study and these previous studies should not be considered inconsistent or contradictory.

There are mixed results in the literature regarding differences in global efficiency from early to late adulthood during resting state. Some have reported that global efficiency decreases with aging [37], while others have reported no significant changes in global efficiency with aging [39–40]. In the current study, while there were no significant average group differences in global efficiency during rest or the 2-back task, there was a significant interaction between age and global efficiency during the 2-back task for predicting individual variability in working memory performance. This interaction between global efficiency and age indicates that the predictive value of network global efficiency is dependent upon the age of the individual. For young participants, slight increases in the global efficiency of the network produce substantial improvements in working memory performance. The ability of the network to effectively integrate information across disparate brain regions facilitates effective working memory performance in young adults. Taking the global efficiency results together, these suggest that there is consistency in the average group global efficiency from rest to task, but the global efficiency scores of individual participants do vary considerably around that mean between rest and 2-back; and critically, the manner in which those individual scores tend to vary around that mean from one condition to the other is systematically related to working memory performance. Our results are consistent with recent work from Stanley et al. (2014), who have shown that a less defined modular structure (and therefore a more globally integrated system) in the entire brain network is associated with improvements in working memory performance even when the number of items that must be held “on line” during the working memory task increases. Thus, the degree of integration in the entire brain seems to be closely related to how well individuals perform on working memory tasks, at least among young adults. Importantly, however, the same magnitude changes in global efficiency among older participants in the current study failed to produce improvements in working memory performance. In fact, increases in global efficiency among older participants were actually associated with slight decreases in predicted working memory performance.

This age-related difference in the predictive utility of global efficiency complements and extends a recently developed model of age-related differences in working memory using a different paradigm. Developed from prior work examining brain activation as opposed to functional connectivity, the Compensation-Related Utilization of Neural Circuits Hypothesis (CRUNCH) maintains that older adults demonstrate comparable working memory performance to young adults during minimally demanding tasks, but that older adults must recruit more brain areas to achieve similar levels of accuracy [93–94]. However, at higher task demands, these mechanisms fail in older adults, resulting in poorer performance and the recruitment of fewer cortical resources in frontal and parietal brain areas; this reflects working memory overload. In the current study, older adults performed considerably worse than younger adults on the 2-back task, suggesting that the task was highly demanding for the older cohort. If the 2-back task were excessively cognitively demanding for the older cohort, then the accompanying recruitment of fewer cortical resources during the task could be associated with the mitigated importance for high global network efficiency to effectively interconnect the relatively fewer cortical areas involved for performing the task. Future work should seek to empirically determine whether the relationship between global network efficiency and the amount of cortical activity observed during working memory tasks changes in accordance with age.

Complex network analyses of neuroimaging data have become increasingly popular, but the meaning of such analyses relative to behavior remains unclear. Is greater global efficiency related to better or worse performance on cognitive tasks? Is greater local efficiency related to better or worse performance on cognitive tasks? Do these relationships change with normal aging? Mapping out these age-related differences in the relationship between whole brain network properties and behavioral performance is especially relevant because decline in working memory can lead to difficulties performing a multitude of everyday activities, and working memory deficits have been associated with both Alzheimer’s disease and Parkinson’s disease [95–97]. The current findings provide an important step toward identifying these relationships.

### Limitations

Differences in the topological properties of the entire functional network have been linked to intelligence [34], motor learning [48] working memory capacity [35], verbal learning and visual recall [36], and increasing working memory load [51]. However, analyses of average whole-brain network metrics may not be sufficient to identify critical differences in network topology between groups because analyzing group averages tends to dilute regional changes and leave localized spatial shifts in network organization undetected [46]. Future work should seek to determine (1) whether localized spatial shifts in network measures can account for individual variability in behavioral performance, and (2) whether whole brain or regional network properties better account for individual variability in behavioral performance
